# Towards Quantitative Spatial Models of Seabed Sediment Composition

**DOI:** 10.1371/journal.pone.0142502

**Published:** 2015-11-23

**Authors:** David Stephens, Markus Diesing

**Affiliations:** Centre for Environment, Fisheries and Aquaculture Science, Lowestoft, Suffolk, United Kingdom; Università di Genova, ITALY

## Abstract

There is a need for fit-for-purpose maps for accurately depicting the types of seabed substrate and habitat and the properties of the seabed for the benefits of research, resource management, conservation and spatial planning. The aim of this study is to determine whether it is possible to predict substrate composition across a large area of seabed using legacy grain-size data and environmental predictors. The study area includes the North Sea up to approximately 58.44°N and the United Kingdom’s parts of the English Channel and the Celtic Seas. The analysis combines outputs from hydrodynamic models as well as optical remote sensing data from satellite platforms and bathymetric variables, which are mainly derived from acoustic remote sensing. We build a statistical regression model to make quantitative predictions of sediment composition (fractions of mud, sand and gravel) using the random forest algorithm. The compositional data is analysed on the additive log-ratio scale. An independent test set indicates that approximately 66% and 71% of the variability of the two log-ratio variables are explained by the predictive models. A EUNIS substrate model, derived from the predicted sediment composition, achieved an overall accuracy of 83% and a kappa coefficient of 0.60. We demonstrate that it is feasible to spatially predict the seabed sediment composition across a large area of continental shelf in a repeatable and validated way. We also highlight the potential for further improvements to the method.

## Introduction

The seabed of the world’s oceans accounts for 71% of the surface of the Earth, harbours significant living and non-living resources, fulfils vital ecosystem services and provides a wide range of habitats for various living organisms. Yet, when it comes to accurately depicting the types of seabed substrate and habitat and the properties of the seabed for the benefits of research, resource management, conservation and spatial planning, it becomes immediately clear that there is a lack of accurate and fit-for-purpose maps and spatial models for most parts of the world’s oceans. The European Marine Observation and Data Network (EMODnet) is an attempt by the European Union to overcome such deficits in European waters. EMODnet assembles marine data, data products and metadata from diverse sources with the purpose to unlock fragmented and hidden marine data resources and to make these available. Among those data types that are being harmonised and made available are bathymetry, seabed substrates and benthic habitats.

Whilst such initiatives to unlock hidden data products and to harmonise these across national boundaries are laudable, it has become increasingly clear that this might be a challenging task. For example, EMODnet-Geology attempts to harmonise seabed substrate maps produced by geological surveys across Europe based on more than thirty differing classification schemes. The resulting classification is hence likely to be the lowest common denominator, with fairly broad classes similar to those proposed by [1]. In any case, maps of seabed substrate will be provided in categorical form (substrate classes) rather than as a quantity. This is somewhat unsatisfactorily, especially if one considers that sediment composition and grain size are of importance for such diverse aspects as benthic community structure [2], *Nephrops norvegicus* burrow densities [3], spatial variation in the abundance of Human Pathogen Indicator Bacteria within estuarine environments [4], permeability in coastal marine sands [5] and compressional velocity of shelf sediments [6].

Alternatively, if data on surface sediment composition (e.g. percentages of mud, sand and gravel) are available, then it should be possible to spatially predict the sediment composition of the seabed across the area of interest. Spatial prediction is the estimation of unknown quantities, based on sample data and assumptions regarding the form of the trend and its variance and spatial correlation [7]. Spatial prediction of environmental properties can be achieved in various ways: Deterministic models such as inverse distance weighted, natural neighbour and nearest neighbour interpolation use observations of a target variable to calculate values at unsampled locations with mathematical functions. Such models use arbitrary or empirical model parameters and don’t provide estimates of model error [8]. Stochastic models incorporate the concept of randomness and provide both estimations of a target variable and an associated error [9]. This class of models includes kriging and regression models among others. In recent years, data-driven machine learning algorithms have become a popular choice. Such algorithms have the advantage that the input data does not need to satisfy strict statistical assumptions as is the case for stochastic models. Machine learning algorithms are flexible statistical prediction techniques that ‘learn’ patterns in data to predict an associated value. Machine learning is defined as “programming computers to optimise a performance criterion using example data or past experience” [10]. As a discipline it falls between computing and statistics and is also referred to as statistical learning. Terrestrial remote sensing has successfully employed these techniques for optical data for several years and they are being used more regularly in seabed mapping using acoustic data.

There is a vast array of statistical learning techniques, including Maximum Likelihood Estimation [11–13], k-Nearest Neighbour [14,15], various decision tree methods [12,13,15–20], Artificial Neural Networks [15,20–22], Support Vector Machines [12,15,20] and Bayesian Decision Rules [15,23], and it would be challenging to incorporate and compare them all in a single study. However, with the exception of a few studies [24,25], such methods have been applied to classification (e.g. prediction of substrate or habitat class) rather than regression (e.g. prediction of sediment composition) tasks. Here, we make quantitative predictions of sediment composition (fractions of mud, sand and gravel) with the Random Forest (RF) algorithm [26], which has become one of the most widely used and successful statistical learning models for classification and regression, showing good performance in a large number of domains [12,14,15,20,25,27–34]. RF is a non-parametric technique, i.e. no assumptions regarding the shape of distributions of the response or predictor variables are made [29]. It can handle complex, non-linear relationships between predictor and response variables.

The aim of this study is to determine whether it is feasible to spatially predict substrate composition across a large area of seabed using legacy grain-size data and environmental predictors. The study combines outputs from hydrodynamic models as well as optical remote sensing data from satellite platforms and bathymetric variables, which are mainly derived from acoustic remote sensing. These variables are tested to determine their contribution to predicting substrate composition. As well as producing mapped layers of best estimate of substrate composition across the study region, prediction intervals are also produced indicating the variability of the conditional distribution.

## Materials and Methods

### Study Area

**Fig 1:**
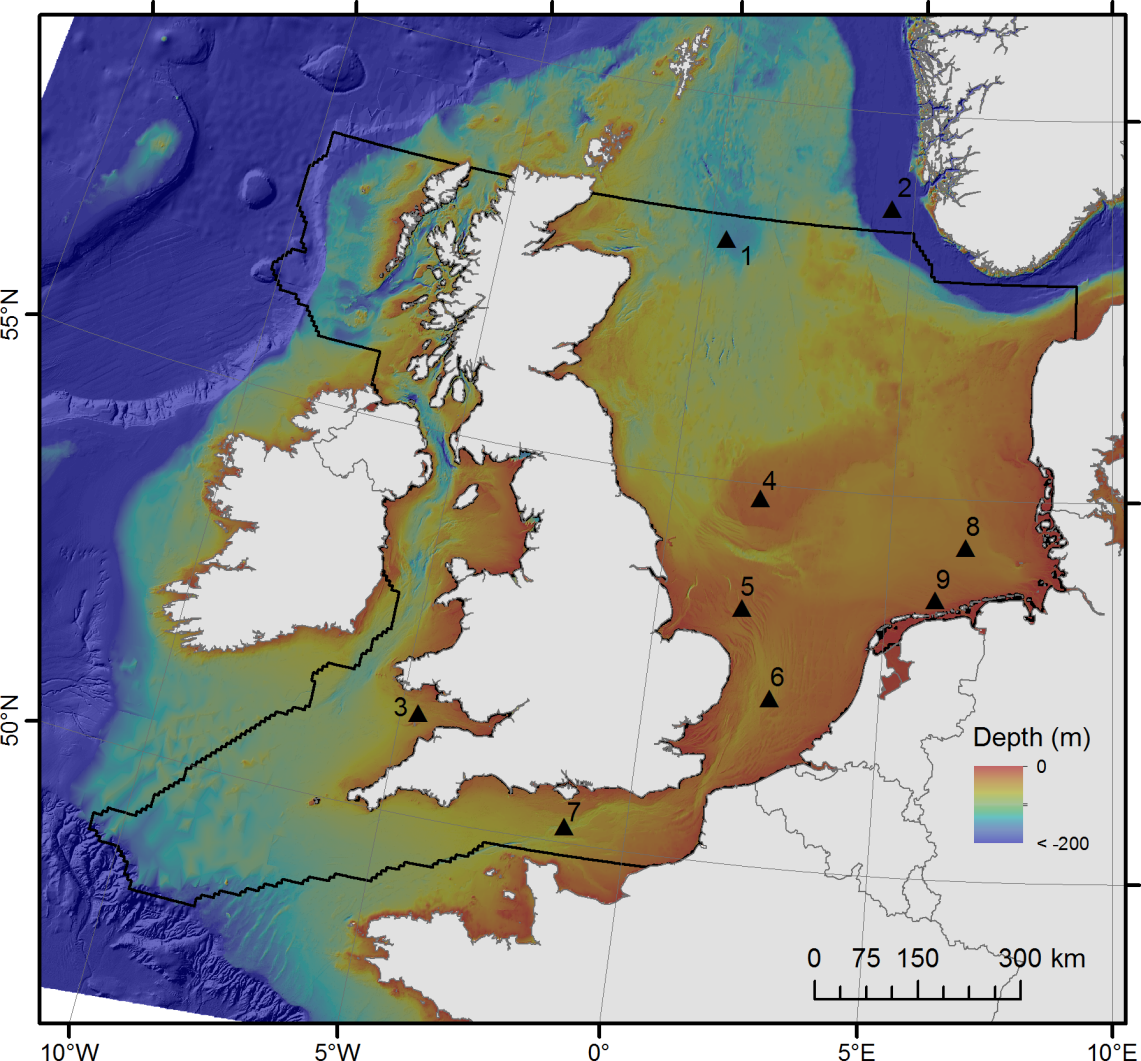
Study area. Strong black line indicates study area. 1) Fladen Grounds; 2) Norwegian Trough; 3) Bristol Channel; 4) Dogger Bank; 5) Norfolk Banks; 6) Southern Bight; 7) English Channel; 8) German Bight 9. Friesian Islands

The study area includes the North Sea up to approximately 58.44°N and the United Kingdom’s (UK) parts of the English Channel and the Celtic Seas (Fig 1). The area covered measures approximately 670,600 km^2^. The water depth extends from 0 m to 2030 m; however the majority of the study area is much shallower continental shelf sea with a median depth of 58 m and 95% of the area being less than 155 m deep.

### Substrate Observations

Substrate observations were collated from a number of sources[35–37] (S1 Table for more details). The minimum requirement for inclusion in this study was that the percentages of the sediment fractions mud (grain size d < 63 μm), sand (63 μm ≤ d < 2 mm) and gravel (d ≥ 2 mm) were reported. Only those observations that intersected with all the predictor variables (see below) were used; this resulted in a total of 57,590 substrate observations (see S1 Fig for the geographic distribution of samples by data source).

### Pre-treatment of response data

The mud, sand and gravel percentages are compositional data i.e. each fraction is part in a total and is constrained between 0 and 1 and the three fractions must sum to 1 (or 100%). Because of this, each component should not be considered in isolation from the others. Here we followed recommendations of Aitchison [38] and transform the data onto the additive log-ratio scale where they can be analysed as two continuous, unconstrained response variables which can assume any value. The outputs of analysis are permutation invariant regarding the denominator [39], here we have chosen to use the gravel fraction.

$$alr_{m}\;=\;\text{log}\left(\frac{mud}{gravel}\right)=\text{log}\left(mud\right)-\text{log}\left(gravel\right)$$

$$alr_{s}\;=\;\text{log}\left(\frac{sand}{gravel}\right)=\text{log}\left(sand\right)-\text{log}\left(gravel\right)$$

The data were then split randomly into training and test datasets. The training data contained 66% of the observations (38,009 observations) and the test set contained 33% (19,581 observations). The test set was used to analyse the prediction performance of the fitted models.

### Predictor Features

Predictor features were initially selected based on their expected importance for explaining the distribution of shelf sediments and their availability. The predictor features comprise digital elevation models (DEM), earth observation data and hydrodynamic model outputs.

A bathymetry DEM for the study area was created from a 6” DEM of the UK continental shelf area [40] and the EMODnet-Bathymetry ¼° (equal to 15”) DEM. Both datasets were merged, whereby the higher-resolution 6” DEM was given priority due to higher resolution, which effectively means that data west of 4°E is derived from the 6” DEM and east of 4°E is derived from the EMODnet-Bathymetry model. The resulting DEM has a cell size of 500 m and is projected in ETRS 1989 LAEA projection. All other predictor features (see below) were resampled onto the same grid. Water depth is considered an important predictor variable, as it is expected to indirectly influence sediment composition, e.g. sediments on shoals tend to be coarser grained than in basins.

Several secondary features were derived from the DEM. These include slope, roughness, rugosity, plan curvature, profile curvature and maximum curvature, all calculated for local neighbourhoods (3x3 kernel). Bathymetric position indices [41] were calculated for neighbourhood sizes of 3, 5, 10, 25, 50, 100, 150, 200, 300, 400 and 500 pixels. The vector ruggedness measure [42] was calculated for neighbourhood sizes of 3, 5, 15, 21, 27, 31 and 33 pixels.

Remote-sensed suspended particulate matter (SPM) were obtained from the MyOcean data portal at a spatial resolution of 1 km. This is algorithmically estimated mineral suspended matter from the MODIS optical sensor [43]. Mean seasonal values for summer (June-July-August) and winter (December-January-February) were calculated between 2002 and 2010. SPM was included in the analysis because certain topographic features (such as the North Norfolk Sandbanks and Dogger Bank) were at least partially visible in the imagery as areas of higher turbidity. Higher SPM concentrations are also found around headlands and areas typically associated with higher current velocities.

Modelled hydrodynamics comprised average current speed and peak orbital velocity of waves at the seabed. These data were calculated for a single year and assumed to be representative. Depth-averaged tidal and wind-driven currents were calculated using the POLCOMS model [44] forced with 15 tidal constituents and hourly wind and pressure at 12 km resolution. Using the same meteorological forcing, the WAM spectral wave model was used to provide the wave orbital velocity at the seabed for the most extreme conditions during the modelling period (denoted as ‘peak orbital velocity’) [45]. Waves and currents cause erosion, transport and deposition of sediments depending on the shear stress they cause at the seabed in relation to grain-size dependent critical shear stresses. They are hence expected to be important direct predictor features for sediment composition.

The Euclidean distance to the coastline was calculated in ESRI ArcGIS v10 and was expected to be an indicator for distance to sediment source (coastal erosion). Table 1 summarises predictor features that were retained subsequent to the feature selection procedure (described below). See S2 Fig for spatial plots of these predictor features.

**Table 1:** Predictor features.

Feature(s)	Description	Unit	Initial resolution	Reference
bathy_500	Bathymetry (water depth)	m	500m	[40]
BPI500_200, BPI500_500	Bathymetric position index. Vertical position of cell relative to neighbourhood (identifies topographic peaks and troughs). 200km and 500km radius used	m	500m	
Eucdist_500	Euclidean distance to nearest coast	m	500m	
spm_S, spm_W	Indicates the amount of inorganic particulate matter suspended in the water column. From MODIS sensor. Mean summer and winter values calculated between 2002–2010.	g/m^3^	1000m	[43]
Av_current	Mean tidal current velocity across water column. Calculated used POLCOM model	m/s	12km	[44]
PeakwaveOrb	Peak Orbital velocity of waves at seabed. Calculated used POLCOM model	m/s	10km	[44]

### Model training

The RF prediction algorithm was chosen as the modelling tool for the analysis because it has shown high predictive accuracy in a number of domains, it is versatile and can be used without extensive parameter tuning, it can handle a large number of predictor features and is insensitive to the inclusion some noisy/irrelevant features. The RF is an ensemble technique developed by Breiman [26]. The algorithm ‘grows’ a large number of regression trees. It is called a *random* forest because two elements of randomness are introduced. Firstly, each tree is constructed from a bootstrapped sample of the training data. Secondly, only a random subset of the predictor variables is used at each split in the tree building process. This has the effect of making each every tree in the forest unique. The underlying principal of the technique is that although each tree in the forest may individually be a poor predictor and that any two trees could give very different answers, by aggregating the predictions over a large number of uncorrelated trees, prediction variance is reduced and accuracy improved [46] (P.316). Observations not included in each tree construction (the ‘out-of-bag’ samples) are then used to create a form of cross-validated prediction error. RF also provides a relative estimate of predictor feature importance. This is a measure of the average reduction in prediction error associated with each variable. The randomForest package [47] in R [48] was used for the implementation of the model. Each forest had 1000 trees, model parameters such as *nodesize* (the maximum size of terminal nodes) and *m*_*try*_ (number of features tested at each split) were kept as default of the package, while some small gains in predictive accuracy can be gained by fine tuning these parameters, typically the improvements are not large.

The Boruta feature selection wrapper algorithm [49] was used to establish predictor features that were significantly important for predicting substrate composition. Wrapper functions identify relevant features by performing multiple runs of predictive models, testing the performance of different subsets [50]. The RF algorithm is a good candidate as a ‘black-box’ for this process because as described previously, it is relatively insensitive to tuning parameters (providing enough trees are grown) and it implicitly produces an estimate of feature importance. The algorithm tests the performance of each predictor feature against introduced random noise features to test whether an increased importance score of that feature is likely to be a ‘real’ or a result of random chance.

The standard implementation of RF aggregates the conditional mean from each tree in the forest to make its ensemble predictions. Rather than just returning the conditional mean, quantile regression infers the whole conditional distribution of the response variable [51]. This is useful when attempting to determine the underlying variability of an estimate and the reliability of a prediction. Substrate composition in some situations will be estimated much more accurately than others. Quantile regression returns prediction intervals, meaning the range in which future observations would be expected to fall, given a certain probability. This should not be confused with confidence intervals, which would typically imply the range in which a population mean is expected to fall). When mapped spatially these intervals could help guide future sampling programs to locations that are predicted with low accuracy. Prediction intervals give an idea of the prediction variability and how confident the model is in its own predictions. The quantregForest package [51] in R was used to calculate 95% prediction intervals. The default parameters of the package were used (nodesize = 10, ntree = 1000).

### Model validation

The RF implicitly carries out a form of cross-validation (CV) using the OOB observations. This usually gives a reliable measure for real model performance assuming enough trees are grown [47]. In addition to this performance indicator, the models constructed here are tested against the test set of observations. For both the CV and the test set, the performance is assessed by calculating the mean of the squared prediction error:

$$MSE_{\hat{y}}\;=\;\frac{1}{n}\sum_{i=1}^{n}{\left(y_{i}-{\hat{y}}_{i}\right)}^{2}$$

Where *y* are observed and $\hat{y}$ are predicted values, The ‘variance explained’ by the models is then calculated by taking the ratio of the MSE to the variance (*σ* ^2^) of the observed values:

$$1-\frac{MSE_{\hat{y}}}{\sigma_{y}^{2}}$$

**Fig 2:**
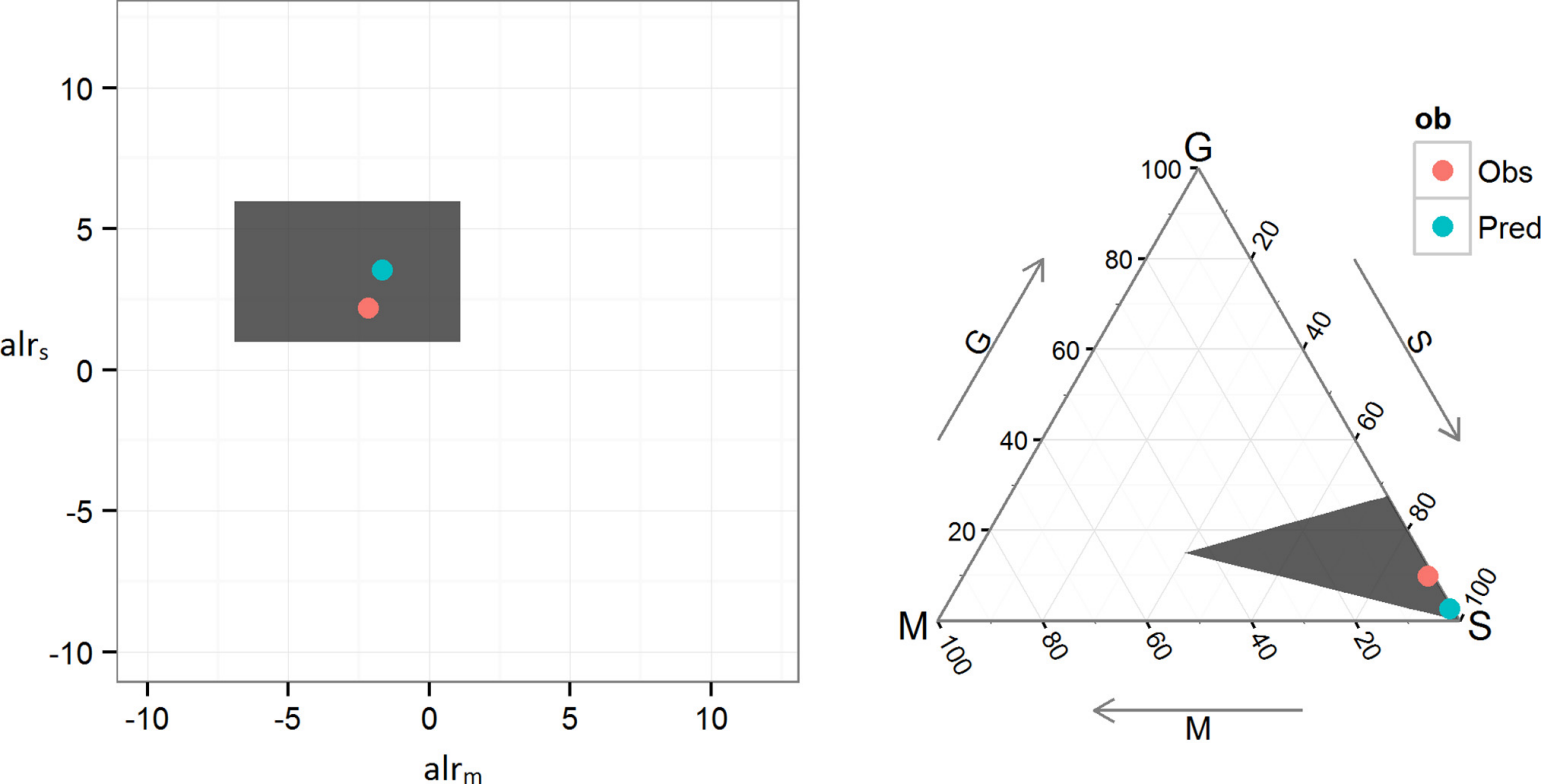
Additive log-ratio transformation. Example of transformation of predictions from log-ratio space to M/S/G fractions for a single test set observation. Predictions are made in the log-ratio space and reverse transformed into M/S/G fractions. The observed value is shown in red, predicted value (conditional mean) shown in green. 95% prediction intervals represented by the grey rectangle.

In order for the predictions to be useful they must be back-transformed from the log-ratios to mud, sand and gravel fractions. This is illustrated for the observed and predicted value of a single test set observation in Fig 2. The 95% prediction intervals appear as a grey window.

Spearman rank correlation index is reported for the observed vs predicted M/S/G values for the test set observations. This is used in preference to the Pearson product-moment correlation which assumes that the variables are approximately normally distributed variables [52]. Also, to give a more intuitive idea of how well the models are predicting substrate at new locations, the test data were transformed into EUNIS substrate classes [1]. These substrate classes provide insight into which substrates the models are predicting effectively and where it is not performing well. Classification accuracy and the kappa coefficient of agreement [53] were calculated from the error matrix.

Finally, we compare classified model outputs against existing high-resolution maps derived from multibeam acoustic data. It cannot be assumed that such maps are accurate, just because they were derived from high-resolution data. Therefore, we only selected maps that fulfilled the following criteria: 1) Maps were derived from multibeam acoustic data and seabed samples reporting sediment composition or class. 2) The mapping process is repeatable, i.e. expert interpretation was kept to a minimum. 3) The maps were validated against an external test set. 4) The maps were published, preferably in a peer-review journal. 5) The maps overlapped with our study site and had a size of at least 1,000 km^2^ to allow for a meaningful comparison. 6) The maps depicted classes that could be derived from the predicted sediment composition (e.g. [1,54]).

The comparison was carried out with the Map Comparison Kit [55], using a ‘per-pixel’ approach, i.e. comparing the class allocations of two maps for every pixel. Therefore, the high-resolution maps were resampled at the resolution of our sediment model. The results of the per-pixel comparisons are reported in a contingency table from which several statistics are calculated. These include the overall agreement between two maps and the kappa coefficient. All data used in the training and validation of the models are included in S1 File.

### Exploration of results

Partial dependence plots [56] (P.369) were generated for the two most important features indicated in model training. They were generated using a random subset of training data. Partial dependency plots indicate the response of the dependant variable across the range of individual features of interest, while averaging out the effects of all other features. They are useful for understanding the nature of the relationship between predictor and response variable, the shape of the underlying function and identifying thresholds in predictor which may be particularly important.

## Results

### Feature selection

**Fig 3:**
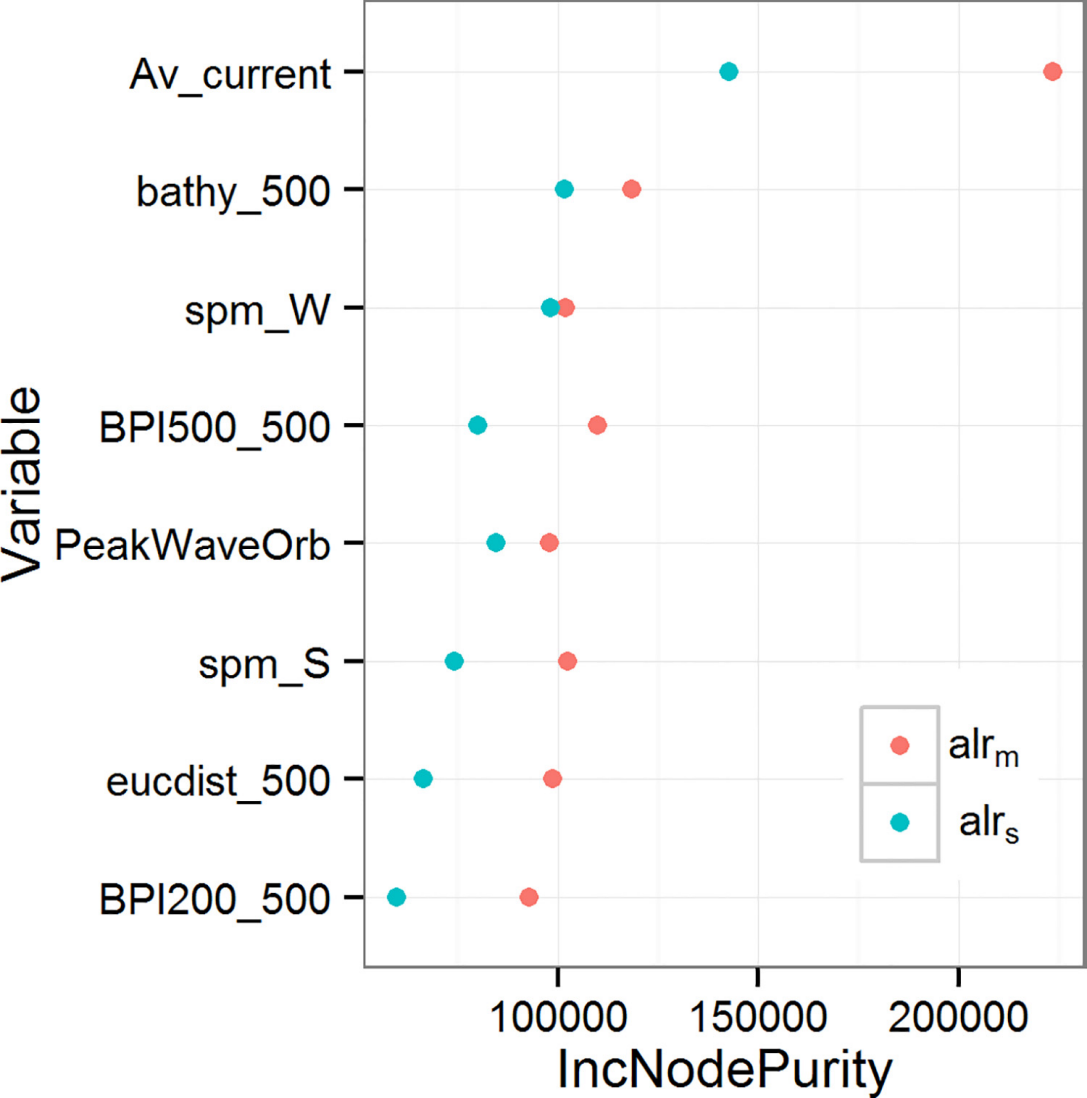
Feature Importance Scores. The importance of predictor features indicated by the random forest algorithm. The x-axis indicates the average decrease in node sum of squares when variable is used.

The feature selection process indicated that all features contributed significantly to the model performance. This would justify training the final model using all the predictor features. However, manual thinning of the features indicated that the CV error rate did not increase when less important features were discarded. The same eight features were identified as most important for both *alr*_*m*_ and *alr*_*s*_. Using these features yielded a CV error almost identical to the model that used all features and so it was decided to use these eight features in the final model (see Table 1). Fig 3 shows the relative importance of the eight features to prediction accuracy.

### Model validation

**Fig 4:**
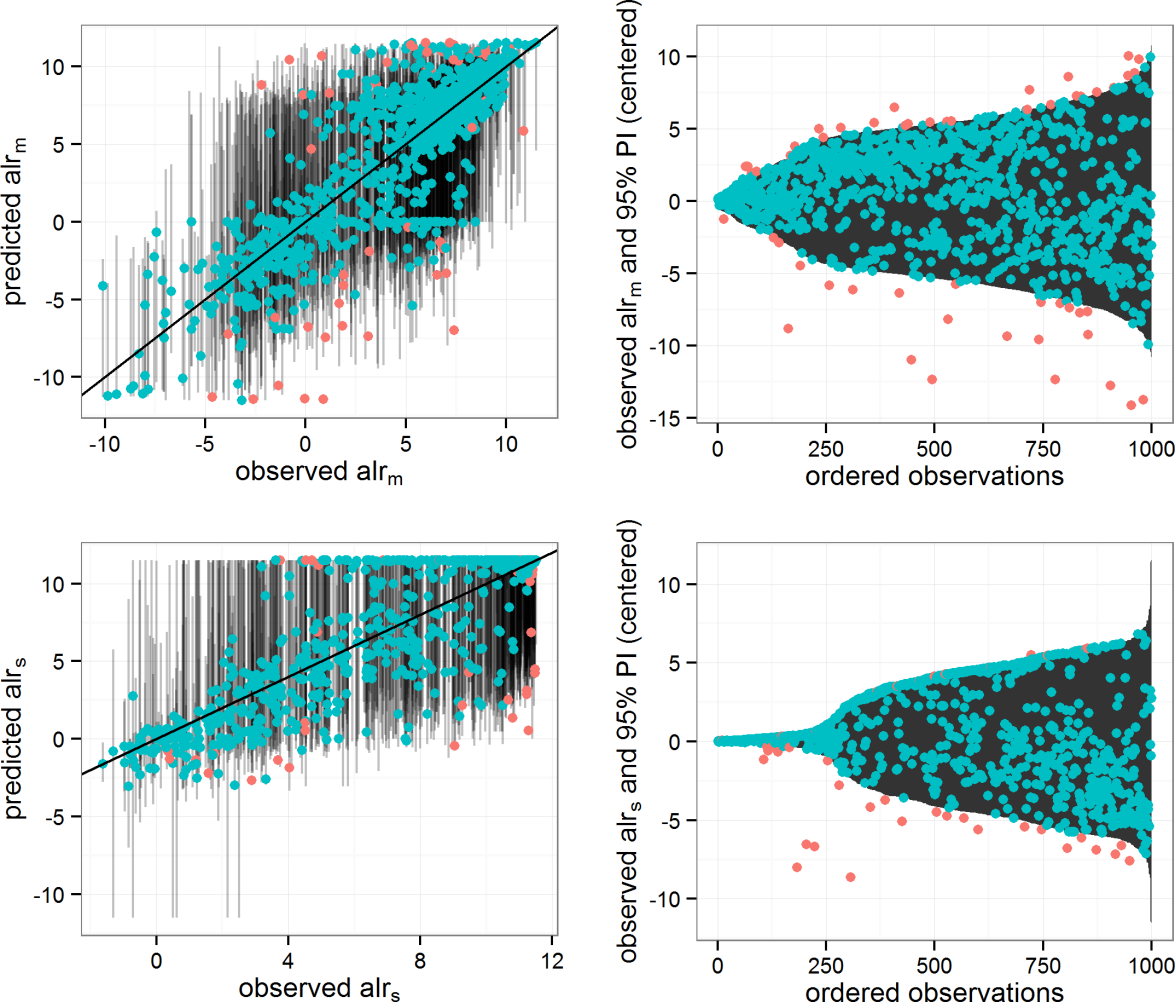
Observed and predicted values for 1,000 random observations from test set. The left panels show predicted vs observed values for *alr*_*m*_ and *alr*_*s*_ along with prediction intervals. The diagonal line indicates y = x. The right panels show the observed values relative to the centre of prediction interval. Points are coloured according to whether the observed value is within the 95% prediction interval.

The model validation statistics (Table 2) indicate that approximately 66% and 71% of the variability of *alr*_*m*_ and *alr*_*s*_ are explained by the predictive models. The close agreement between cross-validated and test set statistics indicates that the models are not ‘over-fitted’ to the training data and that they are generalising the true functions. Fig 4 shows the predictions for 1,000 random observations from the test set. The left panels show observed versus predicted values for *alr*_*m*_ and *alr*_*s*_ along with 95% prediction intervals (black vertical lines). The observed versus predicted scatter plots show a considerable spread in the residuals. The right panels show the observed values relative to 95% prediction intervals. The plots show that the intervals have a high probability of capturing the observed values, 94.3% of *alr*_*m*_ and 95.3% of *alr*_*s*_ observed values are inside the prediction intervals, showing that they are good indicators of prediction reliability.

**Table 2:** Cross-validation and test set performance.

	*alr*_*m*_	*alr*_*s*_
MSE (cross-validation)	9.08	5.84
% Variance explained (cross-validation)	65.82%	70.36%
MSE (test set)	9.24	5.74
% Variance explained (test set)	65.63%	71.00%

Spearman rank correlation for the back-transformed sediment fractions of the test data are as follows: Mud 0.71; Sand 0.75; Gravel 0.78 (p-values for all tests were <0.001).

Observed and predicted sediment fractions were classified into EUNIS substrate types (Table 3), which encompass four broad substrate categories. The confusion matrix shows observed (rows) and predicted (columns) substrate class. The results of the classified test set show a classification agreement of 83% and a kappa coefficient of 0.60. This suggests that the model is performing reasonably well at predicting broad substrate types. However, the error rates for each class show large variability. The lowest misclassification is achieved for ‘sand and muddy sand’ with an error rate of 0.05; however this increases to 0.4 for ‘coarse sediment’, 0.47 for ‘mud and sandy mud’ and 0.81 for ‘mixed sediment’. This indicates that the model is biased towards sandy substrates and is consistently over-predicting the sand fraction. It also highlights the imbalance in substrate types represented in the sampling with the majority of samples falling in the ‘sand and muddy sand’ class.

**Table 3:** Test set EUNIS substrate classification.

	Mud and sandy mud	Sand and muddy sand	Mixed sediment	Coarse sediment	Class error
***Mud and sandy mud***	1229	984	55	32	0.47
***Sand and muddy sand***	252	13039	21	292	0.05
***Mixed sediment***	92	208	126	252	0.81
***Coarse sediment***	27	1088	73	1808	0.40
***Observed agreement***	82.76%				
***Expected agreement***	57.23%				
***Kappa***	0.60				
***Standard error***	0.01	Z = 119.2	P-value < 0.001		
Balanced error rate:	0.43				

Classified model outputs were compared with two high-resolution maps previously produced by the authors: a five model ensemble of substrate type according to a simplified Folk classification [57] and a RF prediction of EUNIS substrates [28]. In the first case (Table 4), overall agreement between the two maps was 83% (kappa = 0.21). However, the relatively high overall agreement is due to a high agreement in the ‘sand’ class, which is dominating the site. Agreement is lower for ‘muddy sand’ and especially ‘gravelly sand’. ‘Sandy gravel’, the least frequent class in the high-resolution map, wasn’t even predicted by our model. These results would suggest that the gravel content is under-predicted by the model. In the second case (Table 5), the overall agreement was lower at 75%, but kappa was higher at 0.34. Contributions to the overall agreement are mainly made by ‘sand and muddy sand’ and, to a lesser extent by ‘coarse sediment’. Agreement between the two maps is very low for the infrequently occurring classes ‘mud and sandy mud’ and ‘mixed sediment’.

**Table 4:** Comparison with high resolution map 1.

Hi-res \ model	Muddy sand	Sand	Gravelly sand	Sandy gravel
***Muddy sand***	406	541	2	0
***Sand***	301	16988	328	0
***Gravelly sand***	2	1897	72	0
***Sandy gravel***	0	244	321	0
***Agreement*:**	82.8%			
***Kappa*:**	0.21			

**Table 5:** Comparison with high resolution map 2.

Hi-res \ model	Mud and sandy mud	Sand and muddy sand	Mixed sediment	Coarse sediment
***Mud and sandy mud***	1	34	0	5
***Sand and muddy sand***	143	5895	16	371
***Mixed sediment***	1	5	0	0
***Coarse sediment***	14	1729	13	1060
***Agreement*:**	74.9%			
***Kappa*:**	0.34			

### Exploration of results

**Fig 5:**
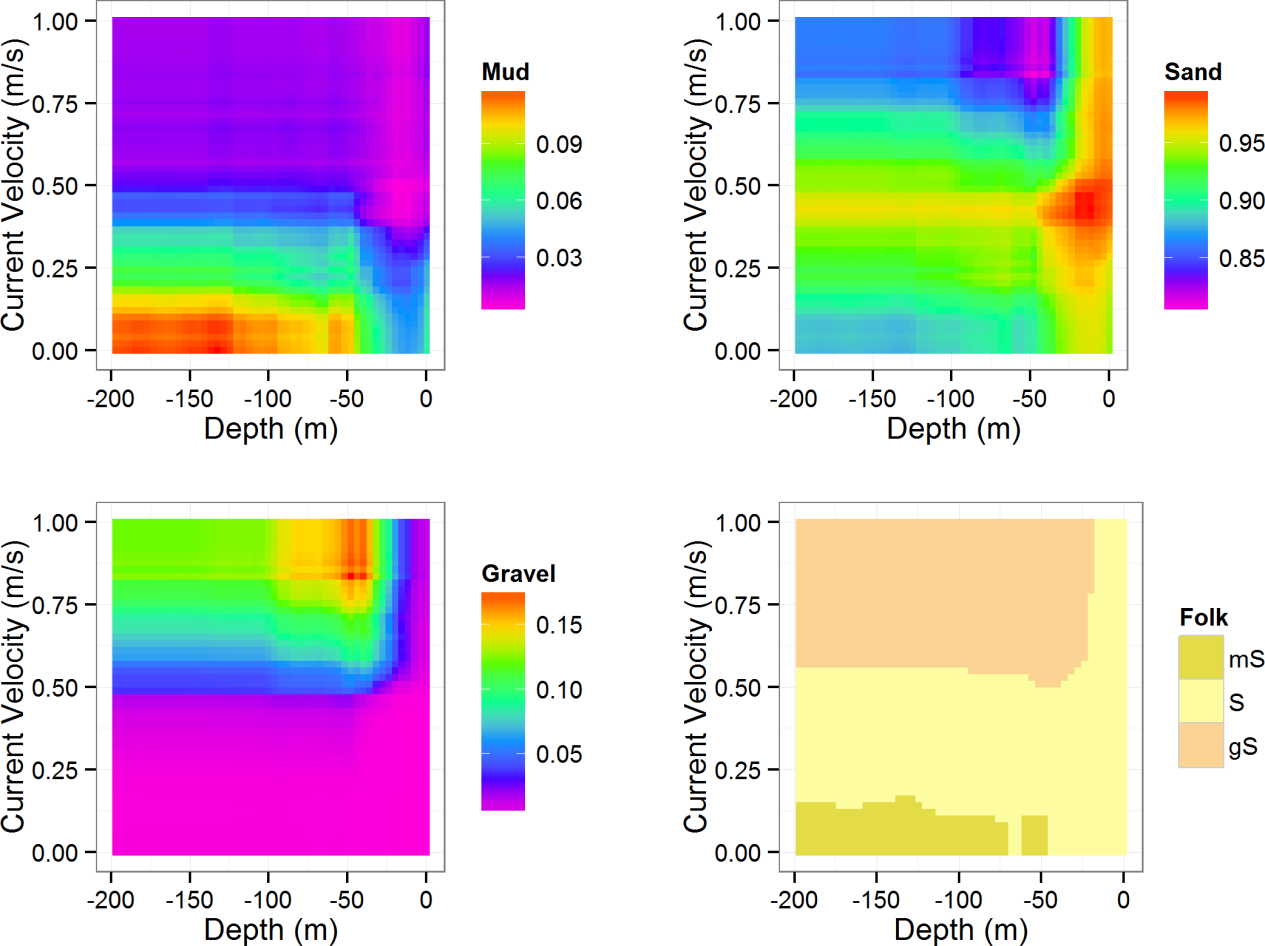
Partial dependence plots. Bivariate partial dependency plots showing response of mud/sand/gravel fractions to water depth and mean current velocity, averaging out the effects of all other variables.

For both *alr*_*m*_ and *alr*_*s*_, average current speed is the most important contribution to prediction accuracy (Fig 3). For *alr*_*m*_ the increase in node purity is considerably larger than for the following features. Bathymetry is the second most important feature for both response variables. Bivariate dependence plots were generated for mean current speed and water depth (Fig 5). The highest mud concentrations (>10%) are located in deeper areas (< -50 m) with low current speeds (<0.25 m/s). Gravel content is highest in moderately shallow areas (around -50 m), where current speeds are highest (>0.75 m/s). Sandy substrates are associated with intermediate conditions (0 to -50 m depth and 0.25–0.5 m/s current speed) and shallow depths, regardless of the current speed. These relationships are mirrored in the fourth panel, which shows simplified Folk classes. Gravelly sands are related to high current speeds (>0.6 m/s) in water depths below -25 m. Muddy sand is most likely to occur below -50 m water depth, where current speeds are low(<0.125 m/s). Sand dominates in the remainder of the diagram.

### Sediment composition and types

**Fig 6:**
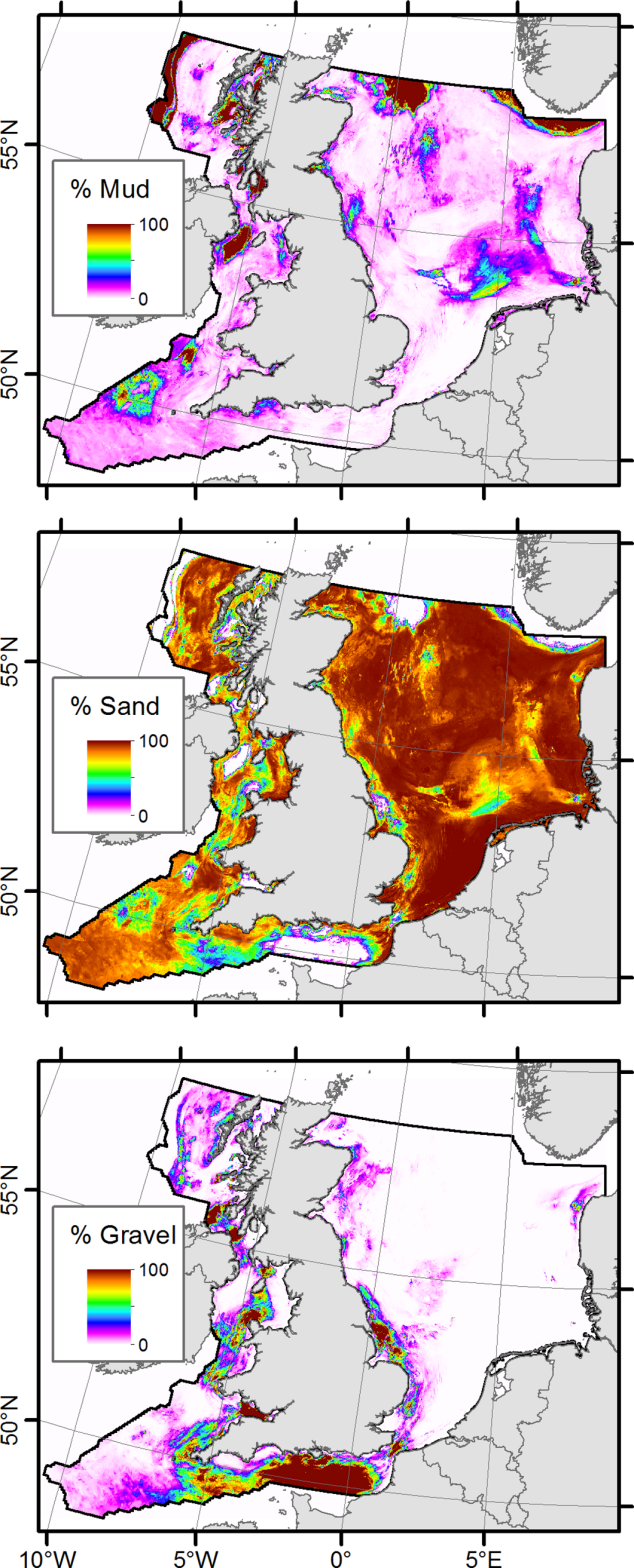
Spatial plots of predicted mud, sand and gravel content.

**Fig 7:**
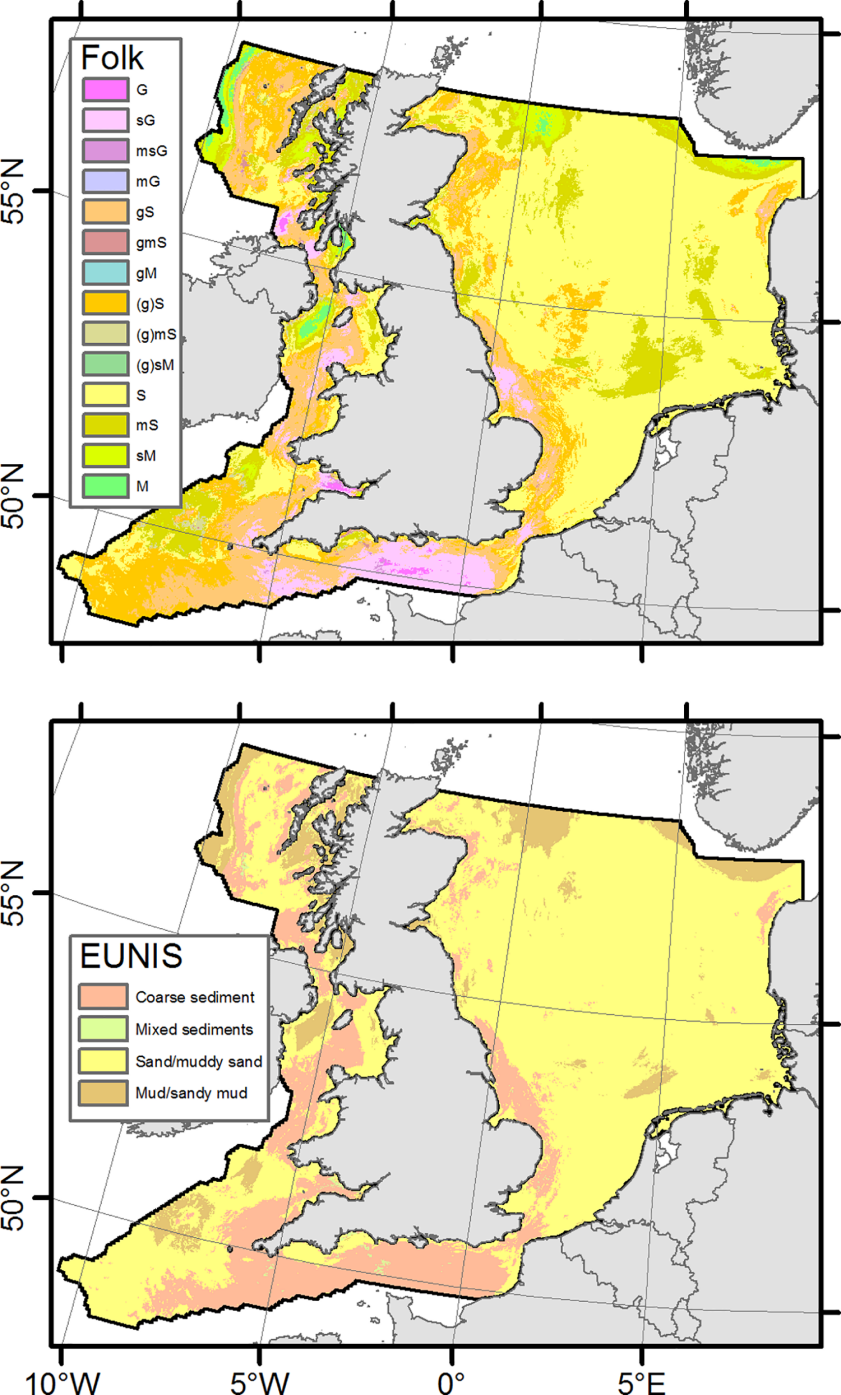
Spatial plots of classified predictions.

Results of the spatial distribution of sediment fractions (mud, sand and gravel) are shown in Fig 6. It is apparent that the sand fraction dominates over large parts of the study area. This is especially the case in the North Sea. The highest amounts of gravel fraction are found in the central English Channel, the Bristol Channel and north of the Irish north coast. Not surprisingly, the locations of maxima in gravel content show a marked correspondence with the locations of tidal-induced bottom shear stress maxima [58]. The highest mud contents are located west of the Scottish west coast beyond the shelf break, on Fladen Ground, in the Norwegian Trough, in the north-western Irish Sea and around Arran Island (-5.2,55.4). The estimates of sediment composition can be easily classified according to EUNIS substrates [1] or simplified Folk textural groups [54]. This is shown in Fig 7.

**Fig 8:**
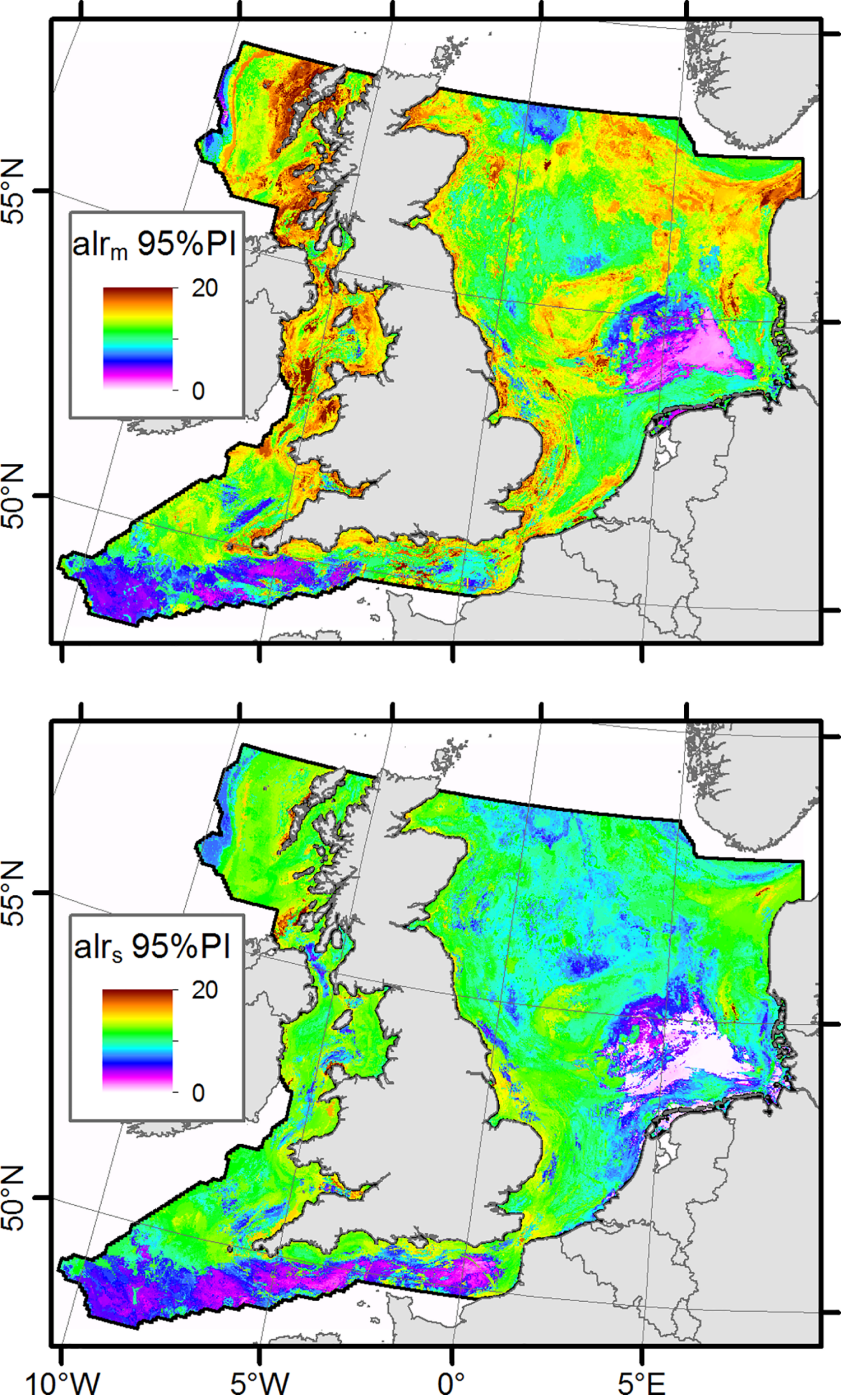
Spatial plots of *alr*_*m*_ and *alr*_*s*_ 95% prediction interval widths.

The 95% prediction intervals for *alr*_*m*_ and *alr*_*s*_ show considerable spatial variation (Fig 8). Although both appear to co-vary across the study site, it is apparent that prediction intervals are generally larger for *alr*_*m*_. Prediction intervals for both *alr*_*m*_ and *alr*_*s*_ are generally low in parts of the German Bight, the English Channel and the Celtic Sea. High prediction intervals are apparent close to the coast, especially around the UK.

## Discussion

### Limitations

Our aim was to predict sediment composition based on legacy grain-size data across parts of the NW European continental shelf. The decision to use legacy data was made out of necessity as it would be unrealistic to collect, process and analyse a sufficient amount of samples over such a large area of seabed. However, this meant that samples had to be obtained from various sources and consequently the data set contains samples that were collected at different times with various sampling gear and analysed according to differing protocols. The BGS dataset for example includes samples that have been collected with 15 different types of equipment (albeit the vast majority with a Shipek grab) between the years 1967 and 1994. It is also known that different grain-size analysis methods produce differing results [59]. Lack of standardisation might lead to increased uncertainty in predictions. Sources of uncertainty might include acquisition method, vintage and timing of sampling, representativeness of subsampling, pre-treatment methods, systematic errors due to particle properties and imperfect conversion models [60]. Harmonisation of data sets would be desirable; however due to incomplete or missing metadata this was not possible.

As indicated above, sampling of the seabed was conducted over several decades. Additionally, the data of the predictor features capture different time intervals, e.g. the hydrodynamics were modelled for the year 2008, whilst SPM estimates were derived for the years 2002 to 2010. An implicit assumption of our models therefore is that the response and predictor variables are constant through time. Principally, such an assumption is unlikely to hold. For example, a decrease in water clarity in the southern and central North Sea over the last 25 years is most likely driven by increased SPM concentrations [61]. Also, waves and currents cause disturbance of the seabed in the study area, with the highest levels occurring in areas of high tidal stress and in shallow regions exposed to waves with large fetch [45]. Such disturbance events might result in net changes in sediment distribution and seabed morphology through time, if net transport of the mobilised sediment is occurring. The influence of waves on the seabed is usually assumed to be limited to water depths less than half the wave length. The wave base, which is the seaward limit of wave influence, typically occurs in depths of 50–70 m around the UK [62]. However, fast (event to inter-annual time scales) morphodynamic adaptations to hydrodynamic forcing conditions are restricted to the upper shoreface [63], which has a depth limit significantly lower than the wave base. It is also known that sediment distribution patterns with high grain-size contrast frequently found in continental shelf settings, might remain stable over years to decades [64–67] despite frequent remobilisation of sediment. Time and space scales of sediment characteristics and forcing functions on continental shelves are inter-linked e.g. [68]. Given the relatively low resolution of our models (500 m pixels), it is unlikely that significant changes in sediment distribution and seabed morphology have occurred within a few decades (equal to the temporal spread of the data).

Predictor features are resolved on various length scales, 500 m for the DEM, 1000 m for the SPM data and 12 km for the hydrodynamic model outputs. These are dictated by various considerations, e.g. limitations to computing power for hydrodynamic modelling and resolution of sourced data sets. It is realistic to expect considerable variability of sediment type within an area of 500 m by 500 m of seabed, but the sediment observations were not sampled at the same scale. The various sampling devices obtain a sample from typically less than 1 m^2^ of seabed; this is the support size of the data and it is different from the support size of the estimates we are making. Averaging quantities over a large area has the effect of reducing the variance and making the distributions more normal [69]. The implications are that it is unrealistic to expect to account for all the variability in our observations when the predictor features have a coarser resolution (larger support size) than the sampled data.

It is likely that there is bias in the model towards the sand fraction. This is a result of the fact that the study is generally dominated by sandier sediments so the sand fraction is overrepresented in the training samples. This means an over-estimate of the sand content at the expense of the mud and gravel contents, as was indicated by the error assessment for the EUNIS substrate map and the comparison with high-resolution maps. It would be worthwhile to investigate methods of compensating for this bias. Possible approaches to explore for mitigating this effect would be to stratify the sampling when building the RF model. This could be done either by substrate class strata so that each tree is built with an equal number of samples from each substrate type. Alternatively, stratifying the sampling spatially could also be an option, this would insure that each tree was built with a sample that was approximately evenly distributed across the study area.

Our model predicts the sediment composition, but it cannot account for rock outcrops at the seabed. Several studies have demonstrated the presence of bedrock at the seabed in the English Channel [70,71], Bristol Channel [72], off Northern Ireland [73,74], the North Sea [28] and elsewhere. However, no estimates on the total area of rocky seabed exist to date. Principally, it would be feasible to predict the presence and absence of bedrock outcrops at the seabed, provided there is a sufficient amount of observations and suitable predictor features.

### Significance of results

We have described a quantitative spatial model of seabed sediment composition for parts of the NW European continental shelf that has been produced with a repeatable method and validated with an independent set of test data. Model validation statistics indicate that our model is performing reasonably well and is not over-fitted to the training data. A EUNIS substrate model, derived from the predicted sediment composition, achieved an overall accuracy of 83% and a kappa coefficient of 0.60. A quantitative comparison with existing high-resolution maps of seabed sediment types demonstrates that the model is reproducing known distribution patterns reasonably well, with overall agreement of 75% and 83%, respectively. To our knowledge, this is the first such model that has been published for this sea area and it is hoped that the results will be utilised by other researchers. The model outputs are downloadable from http://doi.pangaea.de/10.1594/PANGAEA.845468 and might be useful for a wide variety of purposes including, but not limited to, broad-scale habitat mapping, species distribution modelling, hydrodynamic modelling, ecosystem modelling, environmental survey planning, selection of monitoring stations, estimation of other sediment parameters (e.g. porosity, bulk density, total organic carbon) and stock assessments (e.g. *Nephrops norvegicus*). The model outputs are versatile in that they can easily be classified according to different sediment classification schemes, such as EUNIS substrate types [1] or Folk textural groups [54]. This is preferable over classified maps, as different classification schemes might be used for different tasks. However, a Folk map cannot easily be re-classified into a EUNIS substrate map, as the boundary between sandy and muddy substrates is defined as a sand-to-mud ratio of 4:1 in [1] and such a boundary does not exist in the Folk classification.

The presented model belongs to the group of empirical models, which sacrifice generality for precision and reality [75,76]. Because of that, it is considered site-specific and cannot be applied to other areas of the seabed. A dedicated spatial prediction model will have to be built for those areas; however the principal methodology is transferrable. The success of such a model will depend on the availability of suitable target and predictor data sets.

Large parts of the study area have been described as tidally-dominated continental shelf seas, augmented by varying degrees of sub-ordinate storm impact in the North Sea, Celtic Sea and English Channel and fair-weather wave impact in the German Bight [77]. In the southern half of the North Sea, tides dominate the sand transport in most of the Southern Bight and along the east coast of the UK, and storms (stirring by waves and transport by wind-driven currents) dominate in the north-eastern part of the Southern Bight, to the north of the Southern Bight, along the Friesian Islands, in the German Bight and on the Dogger Bank [78]. This is borne out by our model results, in that the feature importance scores (Fig 3) show average current speed as the most important predictor for both *alr*_*m*_ and *alr*_*s*_. The relative importance of storms is also reflected in the variable importance plots, with peak orbital velocity featuring among the eight selected predictor features. With such a strong forcing by hydrodynamic processes in the study area, one might argue that a process-based model, which describes the cause-effect relationships of hydrodynamic forcing and resultant sediment distribution patterns, would be more appropriate to predict the sediment composition of the NW European continental shelf seabed. However, process-based models tend to be realistic and general, but not precise [75], whilst our goal was to make accurate quantitative predictions of sediment composition. Sediment composition of the seabed is a product of multiple processes acting on a wide spectrum of temporal scales from orbital water movements caused by a passing wave (seconds) to glacial-interglacial cycles (100,000 years). In analogy to ecological systems [79] or morphodynamics of coastal systems [80], continental shelf sedimentation and the resulting sediment composition of the seabed are governed by constraints (i.e. laws, such as those describing the mobilisation of sediment by ambient currents) and contingencies. Process-based models would struggle to capture historical contingencies, such as extreme and rare events and geological inheritance. In the particular case, sea-level changes that occurred since the last sea-level lowstand ca. 21,000 years BP [81] have submitted the study area to different sedimentary processes through time and space. Relicts of the past, such as glacial morphological features are still present on the shelf (e.g. moraines, drumlins, flutes and eskers in the northern North Sea and Irish Sea [82–84]) and associated sediments might not be explainable by ambient hydrodynamic forcing alone.

We employed an empirical static model, which assumes equilibrium between the environmental conditions and sediment composition. Arguably, relict features, as mentioned above, and their associated sediments might not be in equilibrium with the ambient hydrodynamic forcing due to the relatively short time span since the submergence of the NW European continental shelf area and stabilisation of the sea level at the current height since approximately 6,000 years BP. However, indirect predictor features such as bathymetry and BPIs might to some extent compensate for this deficit, in that they capture the morphological features that relate to non-equilibrium sediment compositions.

The results indicated by the partial dependency plots (Fig 5) were generally in agreement with expectations, i.e. elevated mud contents associated with deeper sheltered areas, coarser substrates associated with high current speed, and sandy substrates dominating the intermediate conditions. There appears to be certain thresholds of current speed which are associated with boundaries of substrate type, for example 0.125 m/s is approximately the boundary between ‘muddy sand’ and ‘sand’ and 0.5 m/s approximates the boundary from ‘sand’ to ‘gravelly sand’. This relationship is not applicable to shallower areas where ‘sand’ dominates consistently. However, a map of sediment classes derived by applying the above classification rules would only partially resemble the map presented in Fig 7 and misses important features such as Fladen Ground. This indicates that predictor features other than average current speed and bathymetry are also of importance for deriving an accurate representation of the seabed sediment composition.

There appears to be an association between the large prediction intervals and shallower coastal areas, especially around the UK coast. These areas might represent particular conditions which are under-sampled so cannot be predicted accurately. Alternatively, strong environmental gradients and increased temporal variability, which are known to occur in coastal areas, might not be sufficiently resolved in the predictor features. The spatial pattern of the highest prediction interval widths suggests that it is associated with areas of increased hydrodynamic disturbance and this could mean that these areas are inherently more temporally or spatially variable. Large parts of the west coast of Scotland have high PI widths also, these are areas which are highly exposed to Atlantic swell and large parts of this coastline are known to be rocky.

### Potential for improvements

We have demonstrated that it is feasible to spatially predict the seabed sediment composition across a large area of continental shelf in a repeatable and validated way. However, this research has also highlighted the potential for further improvements. These might include:

1. The spatial prediction of rock outcrops across the study area based on rock observations (response variable) and suitable predictor features, such as seabed rugosity and hydrodynamics [85,86].
2. Addressing the imbalance of the sample data set, which is skewed towards sandy substrates.
3. Inclusion of higher-resolution predictor features, especially peak wave orbital velocities, which are likely to poorly resolve processes close to the coast. Also, a higher resolution EMODnet bathymetry data set has recently become available.
4. Extension of the spatial coverage to include the northern North Sea and the Irish and French parts of the NW European continental shelf.

## Supporting Information

S1 Fig Spatial distribution of substrate observations by data source. The number of samples is shown.
(TIF)

S2 Fig Predictor features. (TIF)

S1 File Training and Test data. (ZIP)

S1 Table Data sources. (DOCX)
