# Supplementary figures and images for: Towards Quantitative Spatial Models of Seabed Sediment Composition
Source: PLoS One. 2015 Nov 23;10(11):e0142502. doi: 10.1371/journal.pone.0142502 (PMC4657885; doi:10.1371/journal.pone.0142502)

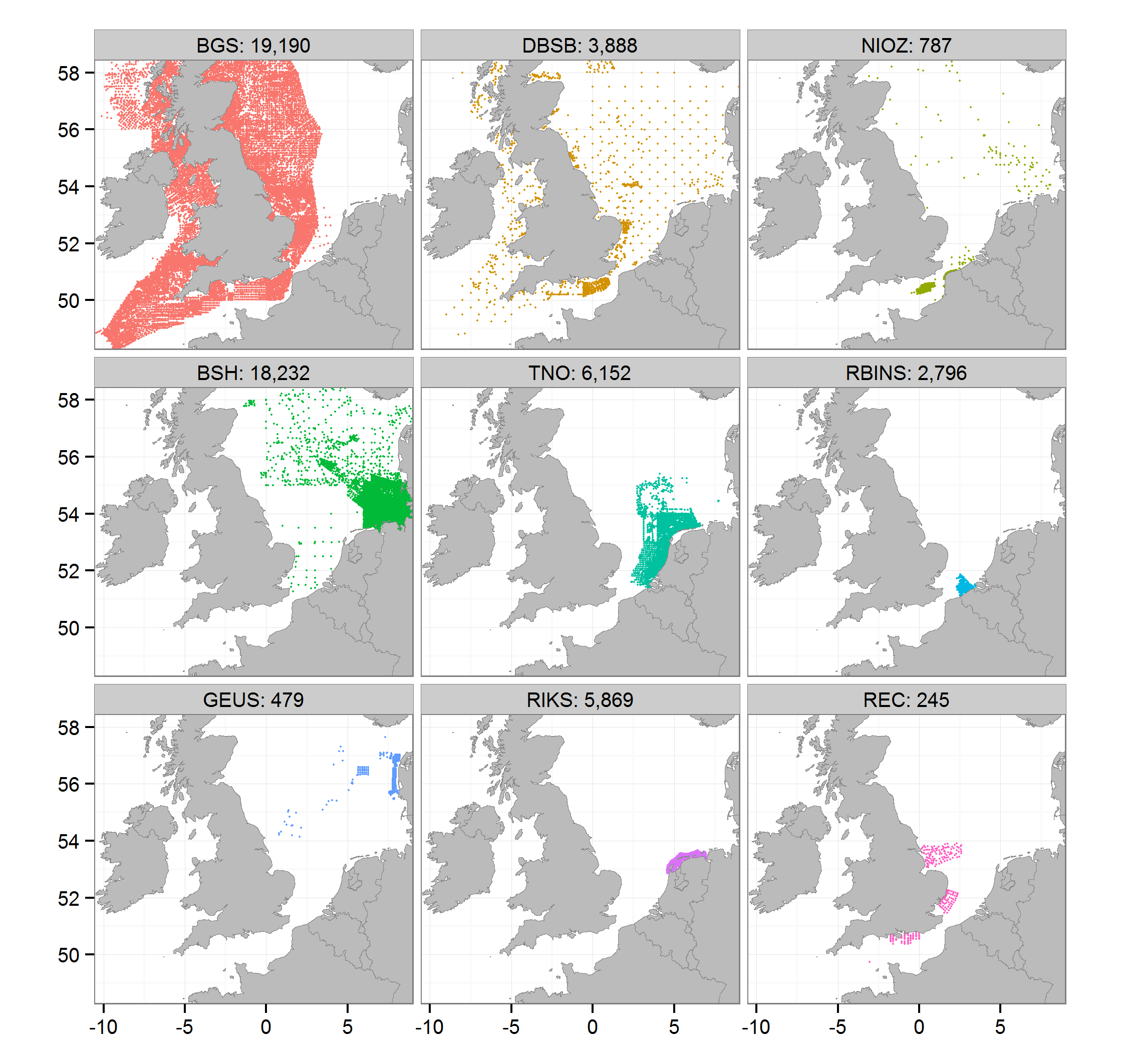

Supplement: S1 Fig — The number of samples is shown. (TIF) [file pone.0142502.s001.tif]

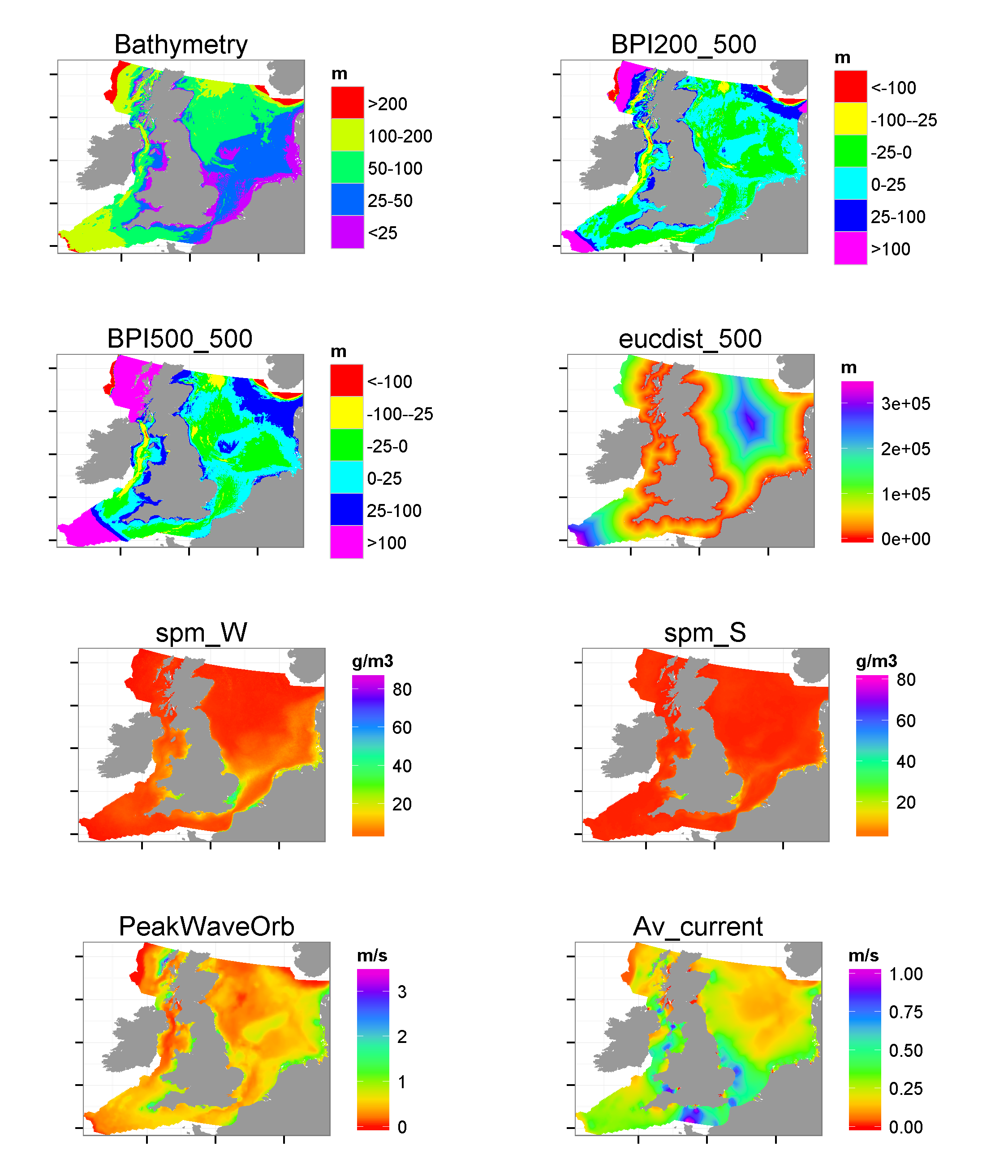

Supplement: S2 Fig — (TIF) [file pone.0142502.s002.tif]
