# Supplementary material for: Towards Quantitative Spatial Models of Seabed Sediment Composition
Source: PLoS One. 2015 Nov 23;10(11):e0142502. doi: 10.1371/journal.pone.0142502 (PMC4657885; doi:10.1371/journal.pone.0142502)
Supplement: S1 Table — (DOCX) [file pone.0142502.s004.docx]

**S1 Table: Data sources.**

| Source | Abbreviation | Number of Samples |
| --- | --- | --- |
| British Geological Survey | BGS | 19,190 |
| dbSeabed | DBSB | 3,888 |
| Federal Maritime and Hydrographic Agency of Germany | BSH | 18,232 |
| Geological Survey of Denmark and Greenland | GEUS | 479 |
| Geological Survey of the Netherlands | TNO | 6,152 |
| Netherlands Institute of Sea Research | NIOZ | 787 |
| UK Regional Environmental Assessments | REC | 245 |
| Rikswaterstaat Netherlands | RIKS | 5,869 |
| Royal Belgian Institute of Natural Sciences | RBINS | 2,796 |
